# Hepatic Encephalopathy in Connection With Budd-Chiari Syndrome due to Infection With Echinococcus Multilocularis: A Case Report

**DOI:** 10.4021/gr306e

**Published:** 2011-05-20

**Authors:** Ahmet Cumhur Dulger, Ozgur Kemik, Fatih Selvi, Huseyin Begenik, Habib Emre, Fatih Mehmet Erdur

**Affiliations:** a Department of Gastroenterology, Medical Faculty, Yuzuncu Yil University, Van, Turkey; b Department of General Surgery, Medical Faculty, Yuzuncu Yil University, Van, Turkey; c Department of Emergency Medicine, Medical Faculty, Yuzuncu Yil University,Van, Turkey; d Department of Internal Medicine, Medical Faculty, Yuzuncu Yil University, Van, Turkey

**Keywords:** Budd-Chiari syndrome, Hepatic encephalopathy, Alveolar echinococcosis

## Abstract

Budd-Chiari syndrome (BCS) is a hepatic venous outflow block generally resulting from disorders affecting hepatic venous system. Elevated hepatic venous pressure results in portal hypertension. BCS may also cause hepatic encephalopathy. Echinococcus multilocularis is a tapeworm parasite and the natural course of the disease may affect liver parenchyma as well as hepatic venous tree. It is the most terrible parasitic disease of the liver and is easily confused with hepatic malignancies. Albendazole therapy may suppress disease progression. Alveolar echinococcosis of the liver rarely causes Budd-Chiari syndrome-related hepatic encephalopathy (HE). We report a rare case of alveolar echinococcosis-related BCS with HE, who was successfully managed by rifaximin and albendazole.

## Introduction

Echinococcus multilocularis (EM) is a cestode parasite and adult worms of the parasite are chiefly found in the small intestines of the carnivores. Humans almost always contribute to the disease cycles as accidental intermediate hosts [1]. The disease is mainly hyperendemic in the entire rural, fox living areas of northern hemisphere as well as in Turkey [2]. Natural course of the disease may go beyond borders of the liver and may cause obstruction of biliary tract as well as hepatic venous system. As a result, the disease mimics a primary malignant liver tumor [3].

Budd-Chiari syndrome (BCS) is characterized by hepatic venous outflow tract obstruction due to a primary venous disease or secondary related to compression or invasion by an adjacent lesion [4]. The patients with worm-damaged hepatic venous system may present with BCS, which gradually leads to ascites, abdominal pain and hepatomegaly [5]. Hepatic failure mostly occurs as a result of massive liver destruction. It is characterized by weakness, jaundice, disorientation, personality changes, flapping tremor (asterixis), prolonged phrothrombin time and hyperammonemia [6].

Alveolar echinococcosis has rarely been implicated in development of hepatic failure. Herein we report a rare case of 57-year-old woman who had E. multilocularis associated BCS which was caused by acute hepatic failure.

## Case Report

A 57-year-old Turkish woman presented to an emergency department with a 7-day history of fever, jaundice, abdominal distention, personality changes, slurred speech, and constipation. The patient was born in a village and lived in the eastern part of Turkey. The patient had a history of hepatic Echinococcus multilocularis, which had been diagnosed 8 years earlier, and she was receiving oral albendazole at a dose of 800 mg per day. At the time of evaluation, she appeared ill. He had a temperature of 38.2 °C, a blood pressure of 80/50 mm Hg, and a heart rate of 86 beats per minute. Her conjunctivas were icteric. There was moderate hepatomegaly with tense ascites. There was no splenomegaly. Her neurologic examination showed flapping tremor (asterixis) and somnolence. The remainder of the physical examination was normal. Blood tests showed a white-cell count of 11,800/mm^3^. An automatic differential cell count revealed 52% eosinophils, 40% neutrophils, and 8% lymphocytes. The hemoglobin level was 10 g/dl, the platelet count was 209,000/mm^3^, the prothrombine time was 17 seconds, and D-dimer level was 5.5 (normal range, 0 to 0.5). The serum sodium level was 125 mmol/L; chloride, 102 mmol/L; potassium, 3.8 mmol/L; bicarbonate, 21 mmol/L; blood urea nitrogen, 10 mg/dl; creatinine, 0.7 mg/dl; glucose, 80 mg/dl; albumin level, 2.8 g/dl; globulin level 4.8 g/dl; aspartate aminotransferase level, 56 U/L (normal range, 0 to 41); alanine aminotransferase level, 48 U/L (normal range, 0 to 40); alkaline phosphatase level, 378 U/L (normal range, 40 to 125); gammaglutamyl transferase level 121 U/L (normal range, 0 to 45) and total bilirubin level, 8 mg/dl (normal range, 0.0 to 0.8 ). Blood ammonia level was 212 mmol/L (normal range 11 - 51) and C-reactive protein level was 48 U/L (normal range 0 - 5). The ELISA test for E. multilocularis was also positive.

**Figure 1:**
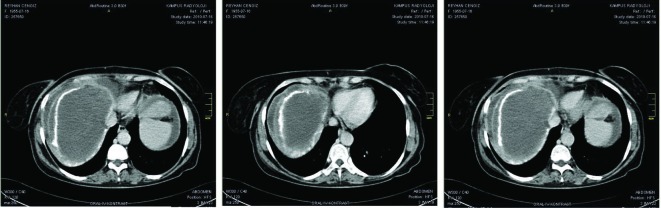
An abdominal CT scan demonstrated multiple hepatic masses in the right lobe of the liver, the largest of which was 12 cm in diameter, non-uniform contrast enhancement of the liver parenchyma, an enlarged caudate lobe, and hepatic and portal vein thrombosis with ascites.

An abdominal paracentesis showed 800/mm^3^ white cell (50% eosinophil); the further examinations in ascitic fluid revealed the glucose level was 87 mg/dl, the protein level was 3.7 mg/dl and the albumin level was 1.5 mg/dl. Serum ascites-albumin gradient was calculated as 1.3 g/dl. At the time of emergency admission, an abdominal ultrasonography showed a hepatic mass, ascites and lack of the visualization of the hepatic veins. An abdominal CT scan also demonstrated multiple hepatic masses in the right lobe of the liver, the largest of which was 12 cm in diameter, nonuniform contrast enhancement of the liver parenchyma, an enlarged caudate lobe, and hepatic and portal vein thrombosis with ascites (Fig. 1). Her echocardiography revealed no major cardiac abnormality. The patient was considered as hepatic encephalopathy due to acute BCS and was given dextrose-containing intravenous fluids with low molecular weight heparin. Ammonia-lowering therapy with rifaximin was also initiated. On day 5 of the patient’s hospitalization, her ammonia level was in normal ranges and clinical parameters were gradually normalized. At this time, albendazole (15 mg/kg per day in divided doses) was also started as adjunctive therapy and she was discharged from hospital with close follow-up.

## Discussion

Human infection with E. multilocularis causing severe Budd-Chiari Syndrome-related hepatic encephalopathy was presented. Alveolar echinococcosis is a zoonotic illness caused by infection with Echinococcus multilocularis which is a cestode fluke. The liver disease in echinococcosis results from the significant destruction of the hepatic parenchyma by the parasitary cysts [7].

Echinococcus multilocularis (alveolar echinococcosis) prevalence varies significantly, depending on geographical location. The disease is hyperendemic in coldest climates of the rural areas, where humans and foxes contact closely, with weak infrastructure. Foxes are definitive hosts of the tapeworm. When dispersed eggs are taken by human, oncospheres are released into the duodenum. They penetrate deep into the intestinal wall and enter the vessels of the portal venous tract, and they usually reach to the liver which is the most suitable organ for producing the hydatid cysts. They also can spread to pulmonary system, brain and skeleton. As part of their nature, these cysts perform an obscure devastating effect on the liver as well as hepatic venous outflow system [8, 9].

The vast majority of cases suffer right upper abdominal pain, jaundice or constitutional symptoms like fatigue or weight loss. The most frequent complications of the disease are biliary cholangitis and sepsis. Indirect laboratory findings of alveolar echinococcosis of the liver include elevation of liver-related transaminases, higher cholestasis enzymes, hyperglobulinemia, eosionophilia and higher C-reactive protein levels [10].

Budd-Chiari syndrome (BCS) is characterized by hepatic venous outflow tract obstruction, regardless of the level or mechanism of obstruction [11]. Cardinal features of BCS include fever, abdominal pain, high gradients ascites, peripheral edema, variceal bleeding, and hepatic encephalopathy [12].

Parasitic and nonparasitic cysts and abscesses may cause compression and thrombosis of the hepatic venous tree. Recent studies have reported a significant but rare association between BCS and alveolar echinococcosis [13-15]. It rarely develops due to compression or invasion of the hepatic veins by the parasitic mass as was seen in the presented case. BCS should be suspected if a patient with a large mass in the liver presents tender hepatomegaly with ascites or fever as was seen in our case. BCS is characterized by high gradient ascites (serum ascites-albumin gradient higher than 1.1) with higher total protein level (higher than 2.5 mg/dl) [5] as was observed in our case. As a striking finding, we also observed an eosinophilic ascites which was compatible with alveolar echinococcosis.

Radiologic techniques remain the gold standard for diagnosis of alveolar echinococcosis of the liver as well as hepatic venous tree. It is reported that ultrasonography should be the first imaging step in the evaluation of the alveolar echinococcosis suspected patient. CT is used as complementary test to ultrasonography. Ultrasonographic findings of BCS are reported to be lack of visualization of hepatic veins, caudate lobe hypertrophy and ascites. CT of abdomen usually shows heterogeneous (patchy) hepatic parenchymal pattern, an enlarged caudate lobe and thrombi in hepatic vein [3]. In the presented case, all of the radiologic findings were compatible with BCS.

Laboratory diagnosis of alveolar echinococcosis is usually made on the basis of serologic tests such as ELISA [16]. Furthermore, on the basis of the radiographic findings, we suspected the diagnosis of echinococcal disease, and a serologic test for echinococcal infection was found as positive.

The liver resection and transplantation are the only ways of treatments that offer the potential for cure, even though only a small minority of cases will actually be cured. Resection should be performed in all patients when eligible. Morbidity is mainly related to variceal bleeding or hepatic failure [17].

The PNM classification for alveolar echinococcosis is recently accepted as an effective method of selecting patients with early-stage alveolar echinococcosis for curative liver resection [18]. According to this classification (Table 1) our patient was in the surgically incurable stage. So we did not perform a surgical approach for the presented case. A long term medical therapy with albendazole can provide palliation in patients with unresectable cysts [19]. In the case under discussion, we started a treatment with albendazole (15 mg/kg in divided doses).

**Table 1:** PNM System for Staging of Human Alveolar Echinococcosis

P	Hepatic localization of the metacestode
PX	Primary lesion unable to be assessed
PO	No detectable hepatic lesion
Pl	Peripheral lesions without biliary or proximal vascular involvement
P2	Central lesions with biliary or proximal vascular involvement of one lobe
P3	Central lesions with biliary or proximal vascular involvement of both lobes or two hepatic veins, or both
P4	Any lesion with extension along the portal vein, inferior vena cava, or hepatic arteries
N	Extrahepatic involvement of neighboring organs
NX	Not evaluable
NO	No regional involvement
N1	Involvement of adjacent organs or tissues
M	Presence or absence of distant metastases
MX	Not completely assessed
MO	No metastases on chest radiograph and CT brain scan
Ml	Metastasis present

Hepatic encephalopathy (HE) is characterized by personality changes with hyperammonemia in patients with known liver disease. It is also defined as a neuropsychiatric disorder resulting from intrinsically impaired synthetic function of the liver. Ammonia remains as the most important laboratory method in the diagnosis of HE. Rifaximin is an oral, minimally absorbed antibiotic and currently accepted as the ideal therapy for HE. It may lower blood ammonia levels by decreasing intestinal bacteria [20, 21].

The patient’s blood ammonia level was higher than normal and flapping tremor was evident. Therefore, we preferred rifaximin (1200 mg/day, divided into 2 doses) for initial therapy of HE.

In summary, we experienced a rare case of HE with BCS due to alveolar echinococcosis. Clinicians should remain vigilant in case of HE especially in rural areas hyperendemic for alveolar echinococcosis.
